# Assessment of the Global Integration Method on the parents’ perception of functional goals in children and adolescents with autism

**DOI:** 10.1590/1980-5764-DN-2024-0154

**Published:** 2025-03-17

**Authors:** Deisiane Oliveira Souto, Amanda Aparecida Alves Cunha Nascimento, Thalita Karla Flores Cruz, Arthur Felipe Barroso de Lima, Gabriela Silva Oliveira, Ana Clara Schaper Fernandes, Vitor Geraldi Haase

**Affiliations:** 1 Universidade Federal de Minas Gerais, Programa de Pós-Graduação em Ciências da Reabilitação, Belo Horizonte MG, Brazil.; 2 Universidade Federal de Minas Gerais, Pós-Graduação em Neurociência, Belo Horizonte MG, Brazil.; 3 Universidade Federal de Minas Gerais, Departamento de Fisioterapia, Belo Horizonte MG, Brazil.; 4 Universidade Federal de Minas Gerais, Departamento de Terapia Ocupacional, Belo Horizonte MG, Brazil.; 5 Universidade Federal de Minas Gerais, Departamento de Psicologia, Belo Horizonte MG, Brazil.; 6 Universidade Federal de Minas Gerais, Programa de Pós-Graduação em Psicologia: Cognição e Comportamento, Belo Horizonte MG, Brazil.

**Keywords:** Autism Spectrum Disorder, Patient Care Team, Clinical Trial, Child, Adolescent, Transtorno do Espectro Autista, Equipe de Assistência ao Paciente, Ensaio Clínico, Criança, Adolescente

## Abstract

Children with autism spectrum disorder (ASD) may experience impairments across all domains of the International Classification of Functioning, Disability, and Health. However, there is a scarcity of programs that integrate interventions encompassing all these domains.

**Objective:**

To investigate parents’ perception of performance and satisfaction with functional goals for children with ASD after intervention with the Global Integration

**Methods:**

This single-group quasi-experimental study involved a total of 98 participants aged 1.8 to 18.2 years who underwent a 3-month intervention, five times a week, for 3-4 h per day. The intervention involved functional task training in an environment inspired by the natural environment associated with the use of a flexible therapeutic suit. The primary outcome measure was the Canadian Occupational Performance Measure, administered before and after the intervention.

**Results:**

Ninety-five participants completed the study. Approximately 60% of participants showed improvements in performance and satisfaction that ranged from 1 to 9 points on the Canadian Occupational Performance Measure. The Global Integration Method program resulted in significant improvements in goal performance (p<0.001, d=0.80) and satisfaction (p<0.001, d=0.67). Most parents (81%) believed that the Global Integration Method incorporated aspects of family-professional collaboration and were satisfied (93%) with the intervention.

**Conclusion:**

In the perception of parents, the Global Integration Method demonstrated effectiveness in achieving functional goals for children with ASD and their families. Parents expressed satisfaction with the intervention and indicated that it incorporated elements of family-professional collaboration.

## RESUMO

Crianças com autismo podem apresentar comprometimentos em todos os domínios da Classificação Internacional de Funcionalidade, Incapacidade e Saúde. Entretanto, há uma escassez de programas que integrem intervenções abrangendo todos esses domínios.

**Objetivo:**

Investigar a percepção dos pais sobre o desempenho e a satisfação de metas funcionais em crianças com autismo após intervenção com o Método de Integração Global.

**Métodos:**

Foi realizado um estudo quase experimental de grupo único. Um total de 98 participantes com idades entre 1,8 e 18,2 anos foram submetidos a uma intervenção de 3 meses, cinco vezes por semana, durante 3-4 horas por dia. A intervenção envolveu treinamento de tarefas funcionais em um ambiente inspirado no ambiente natural associado ao uso de uma veste terapêutica flexível. O desfecho primário foi a Medida Canadense de Desempenho Ocupacional administrada antes e depois da intervenção.

**Resultados:**

Noventa e cinco participantes completaram o estudo. Aproximadamente 60% dos participantes apresentaram melhorias no desempenho e na satisfação que variaram de 1 a 9 pontos na Medida Canadense de Desempenho Ocupacional. O programa Método de Integração Global resultou em melhorias significativas no desempenho das metas (p<0,001, d=0,80) e na satisfação (p<0,001, d=0,67). A maioria dos pais (81%) acredita que o Método de Integração Global incorporou aspectos da colaboração família-profissional e ficou satisfeito (93%) com a intervenção.

**Conclusão:**

O Método de Integração Global demonstrou eficácia no alcance de metas funcionais de crianças com autismo e suas famílias. Os pais expressaram satisfação com a intervenção e indicaram que esta incorporou elementos da colaboração entre família e profissionais.

## INTRODUCTION

Autism spectrum disorder (ASD) consists of a heterogeneous group of neurodevelopmental conditions characterized by difficulties in communication skills, social interaction, and repetitive and stereotyped behaviors, leading to significant functional impairment^1^. Co-occuring conditions, such as intellectual disability, motor and language impairments, sleep disturbances, psychiatric disorders, and genetic syndromes, are frequently observed^2^. The complex and heterogeneous phenomenology of ASD has impacts on all domains of the International Classification of Functioning, Disability, and Health (ICF).

However, programs that integrate interventions at all levels and moderators of ICF are scarce^3,4^. Generally, children with ASD receive speech therapy for their communication difficulties, occupational therapy for their sensory processing deficits, and interventions based on the applied behavioral analysis approach for behavioral and social interaction challenges^5,6,7^. Most children with ASD undergo interventions that predominantly address the socio-communicative symptoms, with impairments at the level of body structures and functions, such as motor alterations, being relatively neglected^5^. An intervention model emphasizing behavioral or communication-related changes may limit access to the multi-professional resources necessary for comprehensive care. Additionally, a persistent challenge in rehabilitation programs is ensuring the transfer of progress achieved in clinical settings to everyday situations. Most contemporary rehabilitation programs are conducted in clinical contexts, where the improvements made do not translate into effective changes in real-life environments. Furthermore, children with ASD and their families often require interventions that are conducted separately and in different locations, further complicating the treatment process for the families involved.

Family-centered practice is the gold standard in pediatric rehabilitation^8,9,10^. Family-centered service constitutes both a philosophy and an approach to service delivery, encompassing a set of values, attitudes, and methods aimed at serving children with special needs and their families^10^. Central to this model is the active involvement of families in the care process to make informed decisions about the services and supports received by the child and family. Family-centered practice involves the active inclusion of families in the care process, taking into account their needs, values, and preferences, as opposed to approaches that do not prioritize this participation^10^. Family-professional collaboration is a strategy to incorporate family-centered practice^9^, characterized by information sharing, open communication, joint decision-making, and integration of family beliefs, needs, and preferences into interventions^9^. This collaborative approach is essential for establishing meaningful goals for the child and family, as well as facilitating the planning and implementation of interventions within the family context. The implementation of this model has been associated with better outcomes for parents and children, such as increased parental satisfaction, improved performance on functional tasks, greater interaction between parents and therapists, and increased family confidence in performing daily routine activities^10,11,12^.

The Global Integration Method (*Método de Integração Global* – MIG) is an intervention program that uses the principles of family-professional collaboration. Consistent with family-centered practice principles, MIG involves shared therapeutic decision-making with the family and also encompasses intervention strategies capable of addressing impairments in all domains of ICF. This integrated, intensive, and interdisciplinary approach was developed for children with ASD based on the best current evidence in pediatric rehabilitation^13,14,15,16,17,18^.

Unlike traditional rehabilitation approaches, care in MIG is provided intensively and interdisciplinary by a single team working collaboratively in one location. MIG is grounded based on the assumption that significant outcomes at the level of body structure and function, *e.g*., involves tissue remodeling and activity-dependent neuroplasticity, which require intense and prolonged intervention^19,20^. The program is proposed to be administered to children for three years, as this is the time necessary for complete tissue renewal^21,22^.

**Figure 1:**
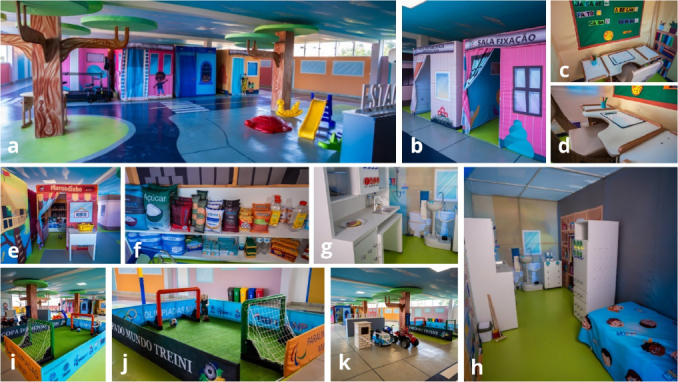
City of Tomorrow. (A) City of Tomorrow Panoramic View; (B) Stimulatory control, controlled instability, and fixation Units; (C) and (D) School Unit; (E) and (F) Market Unit; (G) and (H) Home Unit; (I) and (J) Fitness Space; (K) City of Tomorrow Lateral View.

In MIG, the intensive approach is implemented in an environment called the City of Tomorrow using a flexible therapeutic suit, the “MIG Flex.” The City of Tomorrow (Figure 1) consists of environments inspired by real-life scenarios, such as home, school, market, streets, and sports courts. Through the units of the City of Tomorrow, children have the opportunity to participate in inherently reinforcing activities while developing cognitive schemes and learning to use them in a concrete, contextualized, and relevant way to life. Interventions in children’s living environments or similar settings are believed to promote social learning and generalization^2^.

**Figure 2:**
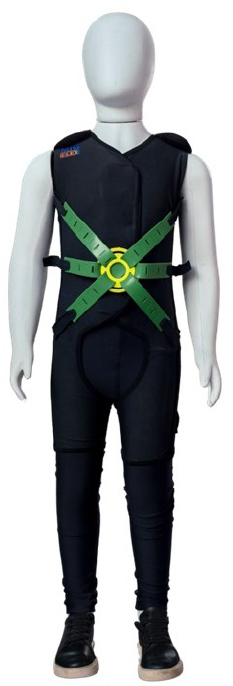
Flexible therapeutic suit (*MIG Flex*).

The activities within the City of Tomorrow are carried out using the MIG Flex (Figure 2). The MIG Flex is based on myofascial meridians. Therefore, the MIG Flex stimulates functional, superficial, lateral, spiral, and deep myofascial lines, providing stability, postural compensation, and active movement with muscle strength enhancement. Stimulation of these lines in an environment inspired by real-life scenarios plays not only a mechanical but also a perceptual role^23,24,25^, contributing to motor skills and environmental exploration. Long-term use of MIG Flex benefits the transmission of correct proprioceptive information to the central nervous system and leads to the active execution of more accurate movements by patients. These benefits contribute to improved cognitive symptoms, as a reduction in motor overload frees processing resources for socio-cognitive learning^26,27^. The use of MIG Flex in the City of Tomorrow promotes active interaction with the environment, fostering gains in skills and competencies during the performance of daily living activities, instrumental activities of daily living, leisure and recreation, and social participation.

To date, no study has investigated the benefits of the MIG program in children and adolescents with ASD. In this single-group quasi-experimental study, the perception of parents/caregivers regarding the performance and satisfaction of functional goals in children and adolescents with ASD was evaluated before and after intervention with the MIG. Furthermore, the study aimed to assess:

- whether the MIG program incorporates aspects of family-professional collaboration as perceived by parents;
- parents’ satisfaction with the MIG program.

It was hypothesized that participants would demonstrate significantly higher perceived performance and satisfaction by parents at the end of the intervention than at baseline.

## METHODS

### Participants

Participants were recruited from 16 clinics located in five different regions of Brazil (South, Southeast, Central-West, North, and Northeast) that offer the MIG intervention program. Children/adolescents who met the following inclusion criteria were invited to participate in the study:

- medical diagnosis of ASD;
- age between 1 and 18 years; and
- classified in support levels 1 and 2, according to the Diagnostic and Statistical Manual of Mental Disorders, Fifth Edition (DSM-5).

Participants with cognitive, behavioral, or clinical limitations preventing them from following instructions and safely participating in activities proposed by the MIG program or those who underwent surgery in the last six months were excluded. A total of 98 children and adolescents, who met the inclusion criteria, participated in the study. The ages of the participants ranged from 1.8 to 18.2 years, with approximately 73.7% being male. All participants were able to communicate verbally. The characteristics of the participants are shown in Table 1.

**Tabela 1:** General characteristics of the participants (mean age 7.14±3.60).

Characteristics	n (%)
Gender	
Male	70 (73.7)
Female	25 (26.3)
Age in years	
1 to 6	53 (56.7)
7 to 12	32 (33.6)
13 to 18	10 (9.7)
Level of support	
Level 1	30 (33.7)
Level 2	52 (56.5)
Not classified	9 (9.8)
Maternal education	
Illiterate or incomplete elementary school	1 (1.1)
Complete elementary school or incomplete middle school	3 (3.3)
Incomplete middle school or incomplete high school	2 (2.2)
Complete high school or incomplete higher education	37 (40.7)
Complete higher education	48 (52.7)
Maternal occupation	
From home	35 (38)
Full-time work	29 (31.5)
Part-time work	28 (30.5)

All parents and guardians signed the Informed Consent Form. Additionally, the children and adolescents, when deemed capable by their parents, also provided their assent to participate in the study. Research procedures were previously approved by the local research ethics committed (CAAE: 72360923.9.0000.5134).

### Instruments

The Canadian Occupational Performance Measure (COPM) was used to define the functional priorities of the participants^28,29,30^. Functional goals are specific therapeutic objectives that are meaningful and relevant to individuals and their family^8,9,10,11^. These goals are considered functional as they are formulated with the purpose of improving the individual’s ability to perform daily activities that are important to them within their life context^20,28^. It is a goal-oriented, individualized, and client-centered tool that involves a semi-structured interview in which parents identify treatment priorities and assess performance levels and satisfaction with the performance on a scale from 1 to 10^29^. During the interview, children and families are invited to list the activities (goals) they believe to be most important and that reflect treatment priorities. Performance on these priorities is scored on a scale of 1 to 10, with 1 indicating unable to perform and 10 indicating able to perform extremely well. Satisfaction with current performance on these priorities is also rated on a scale of 1 to 10, with 1 indicating not at all satisfied and 10 indicating extremely satisfied with current performance. The COPM has been effectively used by children with disabilities and their families and can detect changes in performance over time and after an intervention^30^.

A questionnaire based on the collaborative practice model from An et al.^8^ was adapted to assess family-professional collaboration and family satisfaction regarding treatment through the MIG program. The questionnaire consisted of seven questions, four related to family-professional collaboration and three related to satisfaction with sessions, therapists, and interventions. Each item was evaluated on a 5-point Likert scale, where one corresponds to low family-professional collaboration/low satisfaction levels, and five corresponds to high family-professional collaboration/high satisfaction levels.

### Intervention

Participants underwent three months of intervention, administered up to five times a week, for 3-4 h per day. Three to five therapeutic goals were identified in collaboration with the family, guided by COPM. The interaction between parents and therapists in setting functional goals was characterized by a collaborative approach focused on the specific needs of the child and the family. This collaborative process involves open and continuous communication, in which parents share their expectations, concerns, and observations about the child’s or adolescent’s characteristics, strengths, and weaknesses. In turn, therapists provide their professional perspectives and evidence-based guidance. Together, parents and therapists establish realistic and achievable goals that aligned with therapeutic objectives and integrated harmoniously into the family’s daily routine.

**Figure 3:**
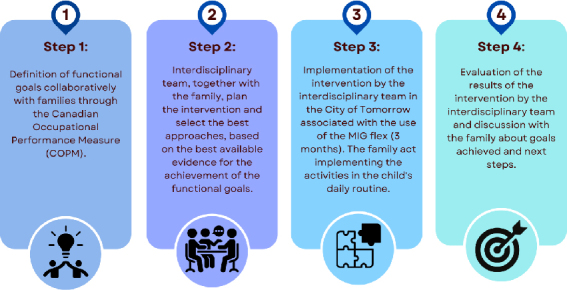
Stages of the *Método de Integração Global* intervention program.

Abbreviation: MIG, *Método de Integração Global*.

Subsequently, an interdisciplinary team developed an individualized intervention plan tailored to the needs of each child. The choice of professional specialties involved in the intervention program and the activities to be developed depended on the specific needs of each participant. The MIG program’s interdisciplinary team comprised physiotherapists, occupational therapists, psychologists, and speech therapists. These specialists provided services at the City of Tomorrow, using the MIG Flex suit as part of the treatment. Each professional applied the best scientific evidence available in their field (Loffi et al.^3^): the physiotherapist focused on motor skills training, the occupational therapist implemented Task-Oriented Training, the speech therapist adopted socio-communicative approaches, and the psychologist employed cognitive-behavioral strategies. The team worked collaboratively to achieve the therapeutic goals defined in conjunction with the children and their families, guiding the choice of therapeutic approaches. Each session of the MIG was conducted by an interdisciplinary team of physiotherapists, occupational therapists, speech therapists, and psychologists. The sessions’ format in the clinics was identical and involved specific task training. This training was carried out using the MIG Flex, in different units of the City of Tomorrow. The intervention was carried out in clinics across different regions of Brazil. All therapists qualified to provide intervention through the MIG program received training involving both in-person and remote training sessions. Additionally, they participated in certification and received ongoing consultations with MIG program specialists. Figure 3 illustrates the steps of the intervention program.

### Statistical analysis

Statistical analyses were performed using the SPSS for Windows, version 22.0^®^ and were subsequently subjected to descriptive analysis with measures of central tendency, dispersion, percentage, and frequency. For descriptive purposes, the goals set by the participants were organized according to the categories of the American Occupational Therapy Association. To test the hypothesis thatparents’ perceptions of their children’s performance and satisfaction regarding functional goals improved following the MIG program intervention, changes in the COPM scores were analyzed. Additionally, a paired sample t-test was used to compare the COPM means before and after the intervention. Cohen’s d was utilized to assess the magnitude of the intervention program’s effects, considering a large effect (d=0.80), medium effect (d=0.50), and small effect (d=0.20). To investigate the hypothesis that the MIG program incorporates aspects of collaboration between family and professionals, and to assess parents’ satisfaction with the program, mean scores and ranges were determined for each question of the collaborative practice and satisfaction questionnaire.

## RESULTS

Of the 98 children and adolescents who started the intervention program, three discontinued treatments due to health reasons. A total of 95 participants completed the 3-month intervention. No adverse effects or discomfort were reported by participants. MIG treatment program’s duration ranged from 12 to 20h per week (17.85±3.2). A total of 425 goals were set by participants, organized, and categorized according to American Occupational Therapy Association categories. Approximately half (46.4%) of the goals were associated with activities of daily living. The most frequent activities of daily living ideintified were personal hygiene (11,9%) and feeding (10%). Instrumental activities of daily living (9.4%), mental functions associated with behavior (9.3%) and social interaction skills (6.4%) were also common goals set by the participants (Table 2).

**Tabela 2:** Participants’ functional goals profile

AOTA categories	AOTA Subcategories	Frequency	(n) %
Daily life activities	Personal hygiene	56	11.9
	Feeding	47	10
	Getting dressed	44	9.3
	Using the toilet and performing personal hygiene	35	7.4
	Bathing and taking a shower	30	6.4
	Functional mobility	11	2.3
Instrumental daily life activities	Communication management	28	5.9
	Establishment and home management	13	2.7
	Community mobility	4	0.8
Body functions	Mental functions related to behavior	44	9.3
	Body functions (attention/concentration)	7	1.5
	Body functions (sensory)	7	1.5
	Neuromusculoskeletal functions	7	1.5
Playing	Exploratory or participatory playing	14	3.0
Social interaction skills	Social interaction with peers and family	30	6.4
Education	Formal education participation	27	5.7
Social participation	Familiar and community participation	4	0.8
Motor skills	Manual abilities and fine motor skills	15	3.2
Rest and sleep	Preparation to rest or sleep	1	0.2
Leisure	Exploratory leisure	1	0.2

Abbreviation: AOTA, American Occupational Therapy Association.

The number of goals that achieved changes in COPM after the intervention is presented in Tables 3 and 4 for performance and satisfaction, respectively. According to parents’ perceptions, 59.6% of the goals showed improvements in performance, ranging from 1 (18.82%) to 9 (0.2%) points. A total of 173 goals (40.07%) showed performance improvement by 2 points or more. The results for satisfaction were similar, with 60.5% of goals showing score improvements after the intervention. Improvements of two or more points in satisfaction were observed in 198 goals (46.5%). All participants showed a positive effect of the intervention on at least one activity, both in performance and satisfaction. The percentages of goals that maintained the same scores in the pre- and post-test were 29.2% and 26.4% for performance and satisfaction, respectively. According to parents’ perception, 48 goals (11.3%) showed a negative effect on performance after the intervention, while negative effects on satisfaction were observed for 56 goals (13.2%). Analyses with Student’s t-test for paired samples revealed significant effects on pre- and post-intervention comparisons, both for performance (t[94]=7.985; p<0.001, d=0.80) and satisfaction (t [94]=6.814; p<0.001, d=0.67).

**Tabela 3:** Number of goals that achieved changes in performance after the intervention.

Intervention effect*	Number of goals	Cumulative number of activities	Cumulative %
<0	48		
0	124	377	88.8
1	80	253	59.6
2	56	173	40.7
3	41	117	27.5
4	37	76	17.9
5	20	39	9.2
6	12	19	4.5
7	5	7	1.7
8	1	2	0.5
9	1	1	0.2

Note: *Change in Canadian Occupational Performance Measure scores.

**Tabela 4:** Number of goals that achieved changes in satisfaction after the intervention.

Intervention effect*	Number of goals	Cumulative number of goals	Cumulative %
<0	56		
0	112	369	86.8
1	6	13	3
2	59	257	60.5
3	40	127	29.9
4	39	87	20.5
5	19	48	11.3
6	16	29	6.8
7	6	13	3
8	5	7	1.7
9	2	2	0.5

Note: *Change in Canadian Occupational Performance Measure scores.

Table 5 provides results on the parent perception questionnaire on family-professional collaboration and satisfaction with the intervention through the MIG program. For items related to family-professional collaboration (1-4), approximately 81% of parents reported large or very large encouragement from therapists during the goal-setting process, intervention planning, and providing information and instructions to implement activities in daily routines. A very small percentage (<2.1%) of the parents reported no encouragement or small encouragement. Approximately 64% of parents reported engaging in activities at home 5-7 days a week to achieve the intervention goals (item 4). Regarding parental satisfaction with the intervention program (items 5 and 6), approximately 93% of parents were satisfied or very satisfied, while only 3% were dissatisfied or very dissatisfied with the MIG program. Finally, 95.2% of parents considered the MIG program to be better or much better than conventional therapies, while 4% considered it equal.

**Tabela 5:** Parent perception about the intervention process.

Questions for parents	Min–Max	Median
To what extent have therapists encouraged you to express your opinion when identifying the goal of your child’s therapy?	2–5	4
To what extent have therapists encouraged you to express your opinion when planning what your child (and family) would do in the therapies?	2–5	4
To what extent have therapists provided information and instructions encouraging you about activities that your child and family can engage in during daily routines to achieve the goal?	2–5	4
How many days per week did your child and family engage in activities to achieve the goal during daily routines?	1–5	4
How satisfied were you with MIG in improving your child’s skills?	1–5	5
Overall, how satisfied were you with the way the therapies were delivered?	1–5	5
Considering your experience with other previously conducted interventions (conventional interventions), how would you describe your experience with MIG?	3–5	5

Abbreviation: MIG, *Método de Integração Global*.

Notes: Response options for items 1, 2, and 3, (5) Very great encouragement; (4) Great encouragement; (3) Moderate encouragement; (2) Small encouragement; (1) No encouragement; Response options for items 4: (5) Everyday; (4) 5–6 days; (3) 4–3 days; (2) 2–1 days; (1) Never; Response options for items 5 and 6: (5) Very satisfied; (4) Satisfied; (3) More or less satisfied; (2) Unsatisfied; (1) Very unsatisfied; Response options for items 7: (5) Much better; (4) Better; (3) Same; (2) Worse; (1) Much worse.

## DISCUSSION

We investigated parents/caregivers’ perception regarding performance and satisfaction of functional goals in children and adolescents with ASD after intervention with the MIG program. Using a pre- and post-intervention single-group design involving 95 participants, we found results that indicated improvements in approximately 60% of functional goals. Parent’s perceptions indicated that MIG incorporated aspects of family-professional collaboration and results also suggest high satisfaction with MIG interventions. This section discusses the main findings of this study.

A persistent issue with rehabilitation programs for children with disabilities is how to promote the generalization of the gains achieved in the clinic to real-life settings^31^. Most current rehabilitation programs are provided in clinics, and the improvements made in these environments do not always transfer to real-life settings. As it is often impractical to implement an intensive and interdisciplinary program in the natural living environments of children, the MIG program suggests simulating these environments through the City of Tomorrow. By simulating a child’s natural environment in the City of Tomorrow, there is a greater probability of generalizing the gains achieved in rehabilitation to natural contexts. This environment, designed based on growing evidence that children learn and succeed better in natural situations and settings^32^, facilitates structured, naturalistic, and active exploration for children with ASD.

ASD poses significant challenges for both families and healthcare professionals, as the necessary interventions for treatment are often provided in a fragmented manner across different locations. This situation makes the treatment process more complex and burdensome for familiesrequiring frequent travel and reconciliation with daily routines. In this context, the MIG program emerges as an innovative solution by centralizing all interventions in a single integrated location. While we recognize that this centralization may not meet all the specific needs of children with ASD, it offers substantial advantages, such as reducing transportation costs and saving time, thereby alleviating the logistical burden on families. Moreover, this integrated approach facilitates access to services and promotes more effective care coordination, which can enhance therapeutic benefits and improve the quality of life for the families involved.

Our results showed changes in performance and satisfaction for approximately 60% of goals, with changes ranging from 1 to 9 points. These findings are consistent with prior studies. Anaby et al.^33^ examined a specific intervention’s effectiveness and found changes that ranged from 1 to 8 points, both for performance and satisfaction (COPM). As in the study by Anaby et al.^33^, in the present study changes of 2-3 or more were observed in most goals. A recent scoping review of 100 intervention studies using the COPM as an outcome measure revealed that 69/100 articles detected a >2 difference in Performance, and 73/100 articles reported a >2 difference in Satisfaction^34^. Although numerous studies consider changes of 2 points on the COPM as clinically significant, this information was not supported by the McColl et al.^34^ scoping review. Nevertheless, this review demonstrated COPM’s ability to detect statistically significant changes over time.

Similarly to data reported in literature, the findings of the present study revealed moderate to large effect sizes for performance and satisfaction results following the intervention. Kim et al.^35^ tested the effects of a family-centered intervention on children with ASD, revealing an effect size of 2.76. Family-focused interventions using the COPM in samples of children with cerebral palsy also found a large effect size (d=3.9)^36^. Additionally, a study examining the effects of a collaborative intervention process found effect sizes of d=0.73 for changes in performance of children with cerebral palsy and parent satisfaction with performance and d=1.08 with post-intervention performance^11^. Thse findings suggest that interventions based on family-centered practices generally yield large effect sizes. It is possible that the large effect sizes found in children’s performance in parent’s perception (COPM) reflect parents’ satisfaction with the family-centered intervention, given the positive association between family-centered practices and parental satisfaction with health services^9^.

The participation of children, adolescents, and their families in the intervention process and goal setting, based on their needs and preferences, are challenging aspects of family-professional collaboration^37^. However, the results of this study suggest that the MIG program successfully incorporates these principles. Approximately 82% of parents perceived that MIG incorporated aspects of family-professional collaboration, demonstrating that this program is grounded in family-centered practice, which is currently considered the best practice in pediatric rehabilitation^8,9^. The collaborative goal setting ensures parents’ participation in defining treatment goals, which is crucial, as families are more likely to engage with goals they consider meaningful^9^. Also, there is evidence that interacting with parents to define goals and treatment plans is highly valuable in parental perception and therapy outcomes, as well as essential for successful programs^8,35^.

The findings of this study highlight significant improvements in functional goals and parental satisfaction, suggesting an effective incorporation of collaboration between family and professionals. The theoretical model of family-centered practices, which underpins the MIG program, emphasizes the importance of active family involvement in the rehabilitation process, promoting collaborative goal setting and the customization of interventions^8,9,10^. This model is supported by theories of situated learning, which suggest that skill transfer is more effective when interventions occur in environments that simulate natural contexts, such as the City of Tomorrow^3^. By aligning with the ICF, the MIG program not only facilitates the generalization of skills acquired in clinical settings to everyday life but also promotes a holistic approach that considers the individual needs and preferences of families. The integration of these theoretical principles may explain the high level of satisfaction reported by parents, reinforcing the effectiveness of family-centered interventions in pediatric rehabilitation contexts. Future research could further explore how these theoretical principles influence parental perception and therapeutic outcomes.

This study has some limitations. Participants were recruited through convenience sampling. Our voluntary sample of parents may have been more motivated than the general population of parents of children with ASD to achieve these objectives. Motivated families are likely to actively participate in the rehabilitation process and replicate the activities developed in clinics in different real-life environments. Since blinding was not possible, expectancy bias may have occurred. Future studies should include randomization and blinding strategies. This study assessed parental perceptions rather than external, objective measures of performance. Future research should consider listening to the perceptions of children/adolescents and using objective instruments to assess performance. No control group was included in this study, which prevented a direct link between the intervention and documented functional improvements in the study participants. In addition to the control group, researchers may also consider long-term follow-up. No follow-up measures were taken, making it impossible to assert whether the gains were sustained beyond the conclusion of the intensive intervention.

Despite these limitations, the findings of this initial study will contribute to substantial advances in ASD rehabilitation. The MIG program aligns with family-centered care and practices focused on different domains of ICF that have been identified as best practices in pediatric rehabilitation^38^. Our results illustrate the importance of collaborative goal setting among parents and therapists for a successful intervention program. The use of the COPM is a valuable tool for collaborative goal setting but should be supplemented by individualized measures that directly assess children and adolescents. Providing an intensive, interdisciplinary therapeutic program in an environment resembling natural settings has demonstrated to be a viable and beneficial strategy for the rehabilitation of children with ASD.
